# Clinical image analysis to build patient-specific models of acute ischemic stroke patients

**DOI:** 10.1007/s13246-025-01646-7

**Published:** 2025-09-29

**Authors:** Virginia Fregona, Ilaria Bottini, Sara Barati, Amedeo Cervo, Antonio Macera, Ghil Schwarz, Guglielmo Pero, Mariangela Piano, Gabriele Dubini, Jose Felix Rodriguez Matas, Giulia Luraghi, Francesco Migliavacca

**Affiliations:** 1 Computational Biomechanics Laboratory – LaBS, Department of Chemistry, Materials and Chemical Engineering ‘Giulio Natta’, Politecnico di Milano, Piazza L. da Vinci 32, 20133 Milan, Italy; 2 Department of Biomedical Sciences, Humanitas University, Pieve Emanuele, Milan, Italy; 3 Neuroradiology Department, ASST Grande Ospedale Metropolitano Niguarda, Milan, Italy; 4 Neurology Department, ASST Grande Ospedale Metropolitano Niguarda, Milan, Italy; 5 Department of Medicine and Surgery, Kore University of Enna, Enna, Italy; 6 Department of Neuroradiology, AOE Cannizzaro, Catania, Italy; 7 Fondazione IRCCS Cà Granda Ospedale Maggiore Policlinico, Milan, Italy

**Keywords:** Thrombectomy, Acute ischemic stroke, In silico medicine, Patient-specific models, Finite element analysis

## Abstract

Mechanical thrombectomy (MT) is an emergency treatment for acute ischemic stroke (AIS) to remove a clot occluding a large cerebral vessel. Histological analysis on retrieved thrombi have shown that they are mainly composed of red blood cells (RBCs), platelets and fibrin, and the outcome of MT appears to be influenced by clot composition. Therefore, being able to predict clot composition from routine medical images used for AIS diagnosis could support the choice of interventional strategy. Along with that, finite element simulations of the MT procedure can help provide insights into the impact of the procedural choices, the vessels morphology and the clot characteristics on the MT outcome. To achieve this, a realistic representation of the involved structures is necessary. In this context, this work aimed to (i) develop a methodology for the analysis of routine radiological images aiming at inferring information about clot characteristics (position, length, and composition) and (ii) develop a semi-automatic pipeline to position the clot in the patient-specific reconstructed geometry to build a patient-specific model which could be the starting point for the in silico replica of the MT procedure. However, image analysis alone could not distinguish between white and mixed clots, while a distinction between red and non-red clots was possible. Consequently, histological analyses were used to assign the clot composition, and thus the mechanical properties, in the positioning simulation. The resulting patient-specific model showed a strong similarity with pre-interventional clinical images.

**Supplementary Information:**

The online version contains supplementary material available at 10.1007/s13246-025-01646-7.

## Introduction

Acute ischemic stroke (AIS) is the third-leading cause of disability and mortality in the world [1], due to the rapid loss of neural tissue when the oxygenation and nourishment of the downstream areas of the brain are impaired by the presence of a clot occluding a cerebral artery [2, 3]. The treatment to remove the occluding clot and recanalize the artery must be rapid and effective [4], and should be performed as early as possible to reduce the possibly lethal consequences (‘time is brain’ [5]). The mechanical thrombectomy (MT), a minimally invasive procedure aimed at removing the clot with the use of stent-retrievers and/or aspiration catheters, was introduced in 2008 [6] and became the standard of care in case of large vessel occlusion (LVO) in 2015, when five randomized clinical trials proved its effectiveness [7–11]. Currently, the rate of substantial reperfusion is 70–80% [12].

The outcome of MT is influenced by the devices and the techniques used, by factors related to the vessel morphology (e.g., tortuosity, presence of occlusions or stenoses upstream of the AIS occlusion, etc.), and by clot characteristics (i.e., clot location, length, and composition) [13, 14]. With the introduction of the MT procedure, retrieved AIS clots became available for histological analysis. The main components of clots are red blood cells (RBCs), platelets, and fibrin fibers [15–20], which are heterogeneously dispersed in the clot [18, 21, 22]. The proportion of the components may vary from patient to patient [21, 22] depending on the etiology of the clot and on the local hemodynamic conditions during clot formation [15]. Clot composition seems to influence the MT outcome [18]; indeed, the RBC-rich (red) clots seem easier to retrieve (fewer maneuvers and shorter procedure times) than fibrin-rich (white) clots [23–25]. Obtaining retrieved clots also enable mechanical characterization [22, 26], which was then extended to clot analogues produced in the laboratory from animal or human blood [27–34]. A general finding in the literature is that increased RBCs content is associated with a decrease in the stiffness of the clot [22, 31, 34].

Since clot composition influences MT outcome, it could be useful for clinicians to predict clot composition from routine radiological exams used in AIS diagnosis, such as non-contrast computed tomography (NCCT), used as a first assessment for discerning ischemic from hemorrhagic stroke [35], and CT angiography (CTA). In the NCCT, the hyperdense artery sign (HAS), corresponding to an increase in x-ray attenuation (measured in Hounsfield Units (HU)), can be assessed. Recently, HAS has been defined as the presence of a segment of an artery presenting a density ≥ 50 HU [36] or when the difference in density between that segment and the contralateral artery is ≥ 4 HU [37]. Studies demonstrated that thrombi showing a HAS contain higher percentages of RBCs than thrombi not showing a HAS [25, 36–38]. CTA uses an iodinated contrast agent to visualize the intracranial blood vessels and roughly identify the proximal end of the occlusion [35]. The acquisition of these images can also be delayed (delayed-phase CTA) to give time to the contrast agent to reach the distal face of the clot through collateral vessels. Identifying a ‘contrast gap’ from the proximal to the distal face of the clot can allow the clinician to predict the clot length [39].

Some studies in the literature investigated the correlation between attenuation values on radiological images and clot composition, both in vitro [40–43] and in vivo [17, 23, 36, 37, 44–46]. Most studies measured the HU in correspondence of the occlusion on the NCCT [17, 23, 36, 40, 41, 44]. Few considered both the NCCT and the contrast-enhanced computed tomography (CECT) [37, 42, 43], and only two included also the CTA [45, 46]. In general, there is not a consensus about the correlation between HU and RBCs content.

As mentioned, several aspects influence the MT outcome. Finite element (FE) simulations of the entire MT procedure virtually performed on patient-specific model, as the one developed and validated by our group [47–49], may be a valuable tool to understand them. In silico models allow, for instance, to evaluate the outcome of the procedure with the same starting occlusion (clot position, length and composition) by changing the devices used or to evaluate how the outcome would change if the clot had different length and/or composition.

This work aims at developing a methodology to semi-automatically create patient-specific models of obstructed patient-specific cerebral vessels realistically reflecting the in vivo pathological conditions before the treatment. This is obtained by (i) developing a consistent and replicable methodology for the analysis of radiological images (CTA and NCCT) with the aim of inferring information about clot characteristics such as position, length, and composition (and, therefore, mechanical properties), and (ii) developing a semi-automatic pipeline to position the clot—with the characteristics found from clinical images—in the patient-specific reconstructed geometry. This will speed up the generation of patient-specific models.

## Methods

Radiological images of seven patients who underwent MT at ASST Grande Ospedale Metropolitano Niguarda (Milan, Italy) were selected from a larger database, based on occlusion characteristics and the availability of complete radiological images. Table 1 reports the availability of the clinical images relevant for the present work for each patient.

**Table 1:** Availability of clinical images for the selected patients

Patient	NCCT	CECT	CTA	3D-CBCT pre	3D-CBCT post
1	Yes	No	Yes	Yes	Yes
2	Yes	Yes	Yes	Yes	Yes
3	No	No	Yes	No	Yes
4	No	No	Yes	Yes	Yes
5	No	No	Yes	No	Yes
6	No	No	Yes	No	Yes
7	Yes	No	Yes	No	Yes

*NCCT* non-contrast computed tomography, *CECT* contrast-enhanced computed tomography, *CTA* computed tomography angiography, *3D-CBCT* three-dimensional cone beam computed tomography, *pre* pre-intervention, *post* post-intervention

### Histological analysis

Thrombotic material collected during MT was promptly fixed in 10% neutral buffered formalin solution, subsequently embedded in paraffin, and sectioned into 2-μm serial slices. The thrombus sections were processed and analyzed according to the standard procedure at ASST Grande Ospedale Metropolitano Niguarda and previously reported [50].

The distinction between red, mixed, and white clot was made with the classification rule suggested by Songsaeng et al. [45]: if the percentual RBCs content outnumbered the percentual fibrin content by more than 20%, the clot was deemed red; conversely, if the percentual fibrin content outnumbered the percentual RBCs content by more than 20%, the clot was deemed white; otherwise, the clot was deemed mixed.

### Image acquisition and analysis

#### CT and CTA acquisition

All NCCT and CTA scans have been acquired in emergency settings with a 64-row scanner (Somatom Definition Edge, Siemens Healthineers, Germany); both were acquired with a volumetric technique with a minimum slice thickness of 0.6 mm. CTA scans were acquired with a multiphasic technique during intravenous injection of 60 ml of iodinated contrast agent (370 mg/ml) [51]. As our standard diagnostic protocol in AIS settings, all patients also underwent a brain perfusion CT, which has not been used in this specific retrospective study.

#### Endovascular procedure

All the MT endovascular procedures were conducted under conscious sedation via femoral arterial access. In all procedures, a stent-retriever-assisted vacuum-locked extraction (SAVE) technique was employed, combining distal aspiration using a large-bore catheter positioned in front of the clot with the retrieval of the clot using a stent-retriever.

At the end of the procedure, a three-dimensional cone-beam CT (3D-CBCT) of the intracranial arterial tree was obtained, providing a complete patient-specific vascular model for the subsequent in silico analysis.

#### Clot location and approximate length

3D-CBCT and maximum intensity projection (MIP) reconstructions at 24 mm of the CTA were used to determine clot location. From post-intervention 3D-CBCT, the geometry of the vessels of interest (ICA, proximal ACA and MCA up to the bifurcation of the M2s into the M3s was obtained through segmentation with the software Horos (Horos Project), setting a pixel intensity threshold between 2500 and 3500. In case the pre-intervention 3D-CBCT was also available, the vessels up to the proximal end of the occlusion were segmented using the same procedure. Then, the two 3D geometries (pre- and post-intervention) were superimposed to identify the position of the proximal end of the clot; however, precise information about the position of the distal end of the clot could not be obtained.

**Fig. 1:**
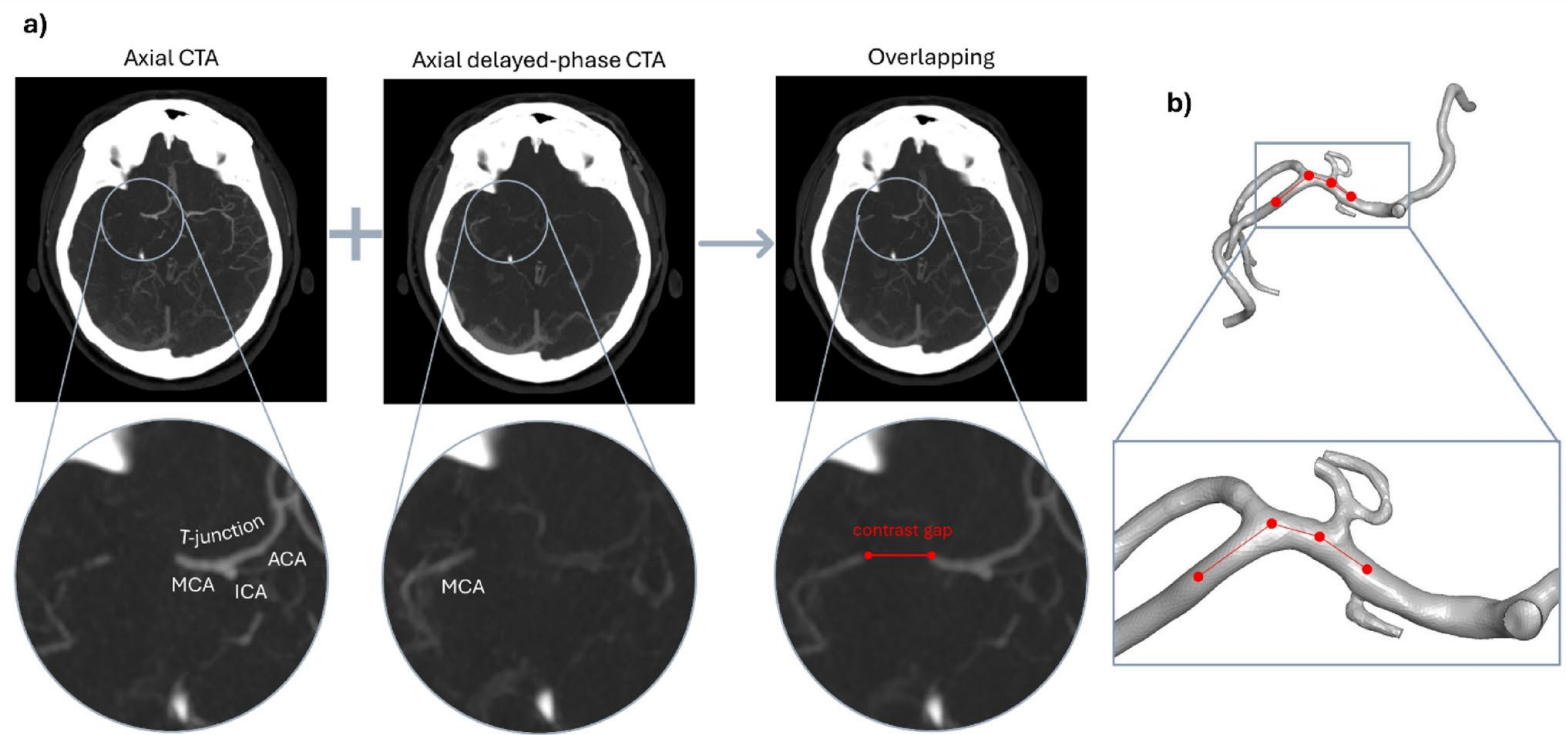
**a** Axial CTAs (left) are overlapped with the respective late-phase CTAs (middle) to visualise the locations of both proximal and distal end of the contrast gap (right). Under each image the respective enlargement of the occlusion area is reported. **b** Contrast gap length obtained on the segmented vessels

For all patients, CTA and late-phase CTA images were used to determine the clot length; they were co-registered and overlapped using the software 3D Slicer (Bringham and Women’s Hospital, Harvard University and NIH, USA) to visualize the ‘contrast gap’ in correspondence of the occlusion (Fig. 1a). This distance can be measured on the overlapped images to estimate the clot extension (Fig. 1a, right). However, assuming a straight path from the proximal to the distal face of the clot may result in an underestimation of the actual length of the contrast gap. To address this problem, the same measurement was performed on the segmented vessel geometry (Fig. 1b).

#### Clot composition

**Fig. 2:**
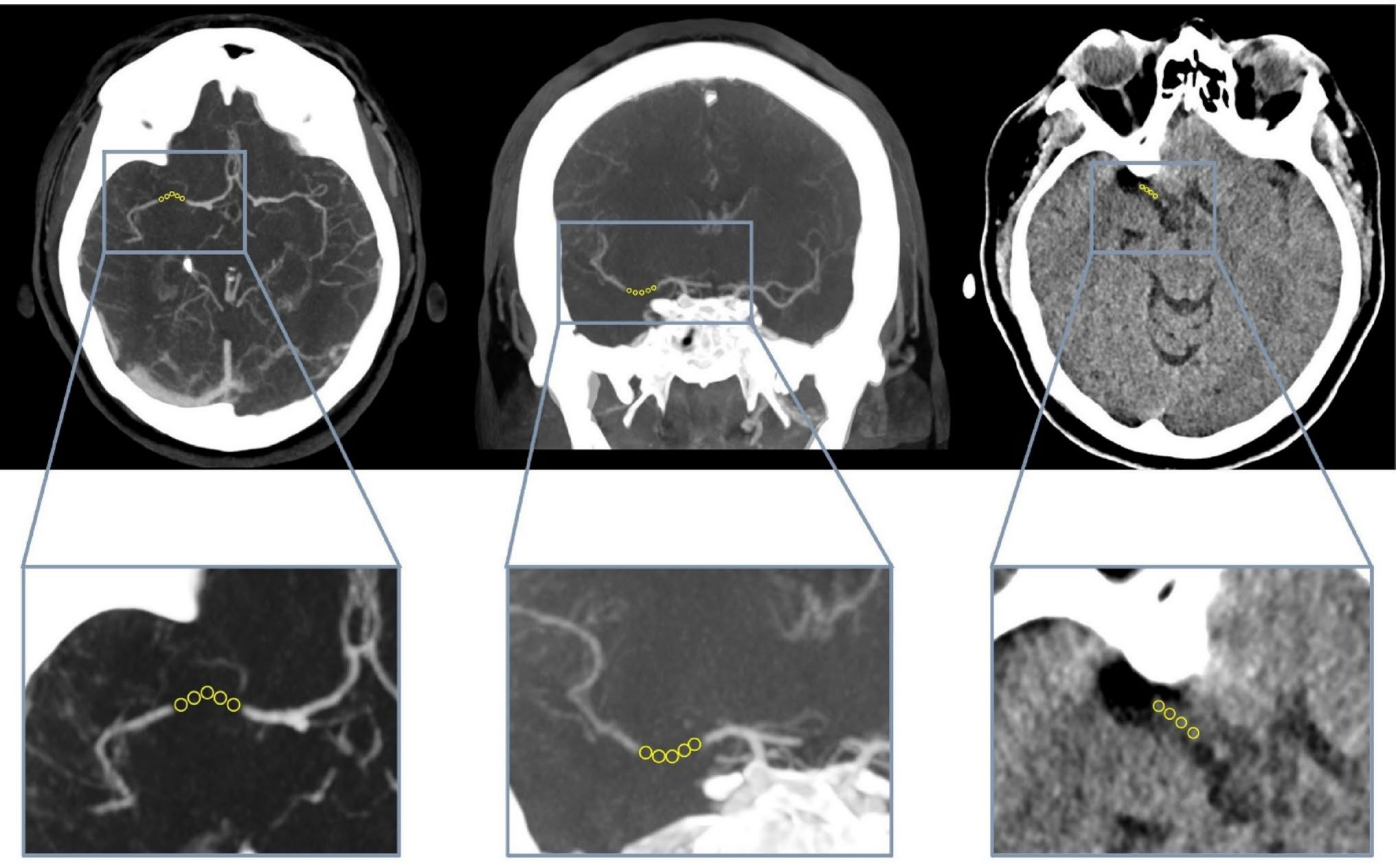
Examples of ROIs on axial (left) and coronal (middle) views on overlapped CTAs. ROIs on axial NCCT image (right)

To determine clot type (i.e., composition), five small circular regions of interest (ROIs) were identified within the clot on the NCCT, CTA, and, if available, the CECT, and the median density value for each ROI was calculated using 3D Slicer (Fig. 2). Finally, the median of these five values was compared with the density values found in the literature (Table 2).

**Table 2:** HU values for red, mixed, and white thrombi on NCCT, CTA, and CECT

HU values									Reference
NCCT			CTA			CECT			
Red	Mixed	White	Red	Mixed	White	Red	Mixed	White	
72.0–89.0	–	31.0–38.0	–	–	–	–	–	–	Cruts et al. [42]
73.5–77.5	56.0–63.0	50.5–59.0	75.25–81.5	52.0–68.0	52.0–66.5	73.75–77.5	54.75–65.75	50.0–60.5	Songsaeng et al. [45]
59.4–69.8	30.9–35.9	29.4–31.0	–	–	–	–	–	–	Ding et al. [41]

–Data non present

### FE simulation of clot positioning

**Fig. 3:**
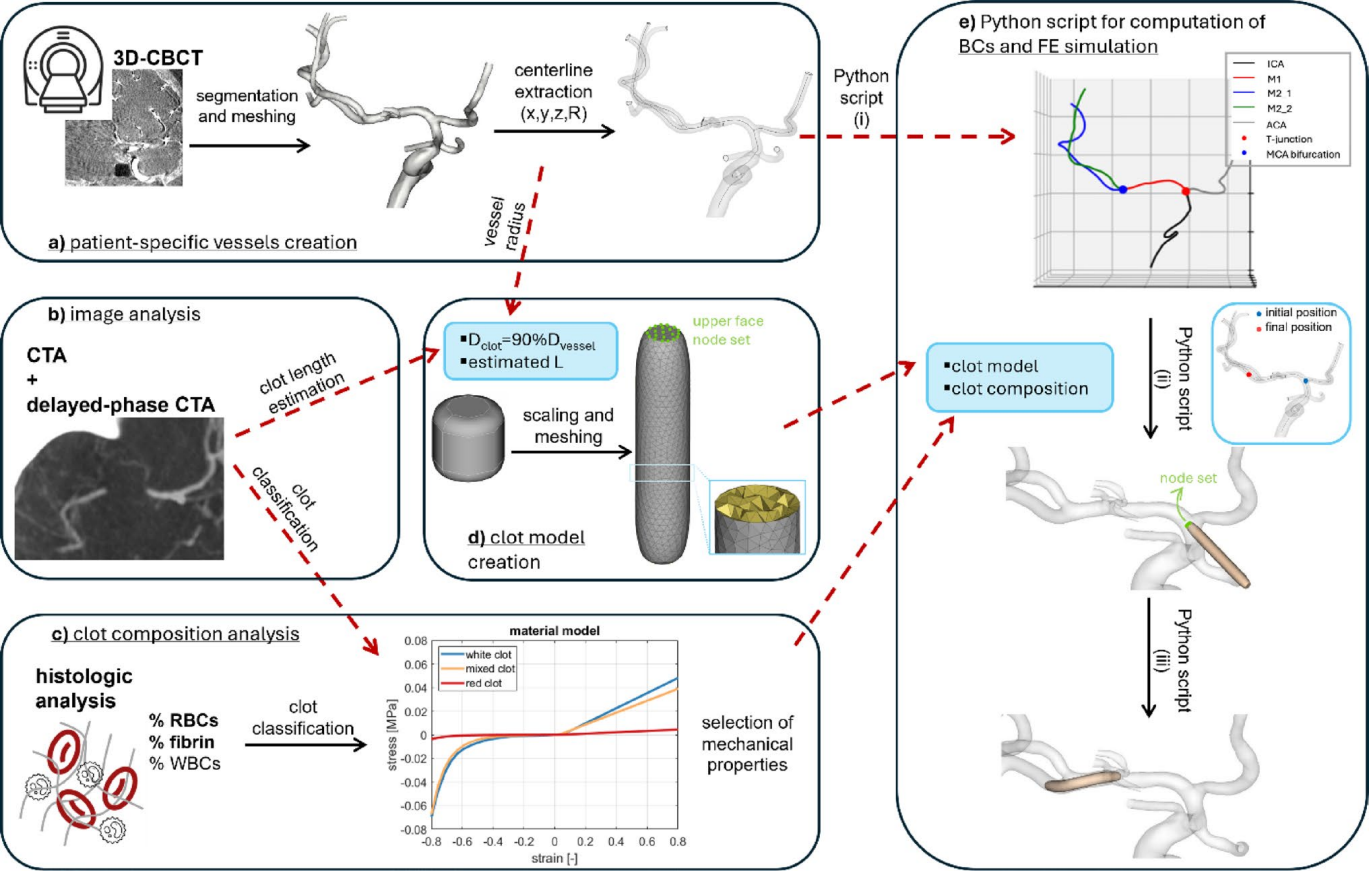
Schematic representation of the workflow, where the red arrows show the flux of information. **a** Patient-specific vessels are segmented from 3D-CBCT, and the centreline is extracted. **b** CTA and late-phase CTA images are overlapped to estimate the clot length and clot composition. **c** Histologic analysis is performed to quantify the content of RBCs, fibrin, and WBCs in the retrieved clots. **d** Clot length and vessel radius are inputs for the Python script compiled in ANSA for the creation of the clot model starting from the unitary clot. A node set of the upper face of the clot is created. **e** The centreline of the vessels is given as input for the second Python script. Each vessel is recognized. Then the user can define initial position for the FE simulation of the clot positioning, and the boundary conditions for the node set of the distal face of the clot are computed according to the final position chosen by the user. Then the simulation is run and the patient-specific model of the patient is obtained

If the vessels segmented from the post-intervention 3D-CBCT presented vasospasm, it was corrected in Meshmixer (Autodesk, CA, USA) to restore the original diameter of the vessels. Then, the geometry was discretized in ANSA (BETA CAE Systems, Switzerland) with 0.3 mm triangular shell elements. The vessels were modeled as rigid. The coordinates of the points discretizing the centerline of each vessel, and the corresponding vessel radius, were extracted using the software VMTK (Orobix s.r.l.), and sampled at 0.5 mm (Fig. 3a).

The clot was modelled as a cylinder beveled at the extremities whose diameter and length were set depending on the patient’s data. The length was the one estimated from clinical images (“Image acquisition and analysis” Section), while the diameter was chosen to occlude 90% of the vessel lumen. A reference clot centered at the axis origin, aligned with the z-axis, and with both diameter and length of 1 mm and a fillet radius of 0.2 mm was created in Solidworks (Dassault Systèmes, Vélizy-Villacoublay, France). A first Python script to be compiled in the ANSA environment scaled the geometry to the desired diameter and length. Then the geometry was discretized with 0.2 mm tetrahedral elements and created a set containing all the nodes belonging to the upper face of the clot (Fig. 3d).

A second Python script (Fig. 3e) computed the initial position of the clot, and the position to replicate the in vivo occlusion. The script was composed of three parts: (i) recognition of the different branches from the reconstructed patient-specific domain (ICA, MCA, ACA), (ii) selection and computation of the initial position of the clot, (iii) selection of the final position of the clot and computation of the boundary conditions to reach the selected position. The scaled and meshed clot model and the vessel centerline must be given as input to the script. In the first part (Python script (i) in Fig. 3e), the different vessels composing the model were recognized based on their length, being the ACA always cut during segmentation to be shorter than the MCA. Once the vessels had been recognized, also the T-junction and the MCA bifurcation were identified. In the second part (Python script (ii) in Fig. 3e), a point on the centerline was selected as the initial position of the clot. This point was selected at the T-junction or in the proximal segment of the ICA if the occlusion was in the MCA or at the ICA apex, respectively. The vector connecting that point with the subsequent one on the centerline was computed and used to compute the rotation matrix to rotate the vector oriented as the axis of the clot (z-axis) to align with the centerline vector just computed. Applying this rotation matrix, the clot was rotated, and the new coordinates of its nodes were computed. Then, the translation vector generated between the central node of the upper face of the clot and the starting point of the centerline was used to translate the clot. Again, the new coordinates of its nodes were computed, and the rotated and translated clot model (initial configuration of the simulation) was saved. Finally, in the third part (Python script (iii) in Fig. 3e) the target point was selected based on the image analysis performed before (“Image acquisition and analysis” Section), as the point in correspondence with the distal end of the contrast gap. The target point was used to compute the translational boundary conditions to be assigned to each node contained in the node set defined in ANSA (upper face of the clot). To do that, the same process of computing the rotation matrix and translation vector used before was here used iteratively for each node of the vessel centerline to align the normal of the distal face of the clot to the centerline, and to translate it from one point to the next one. Each time the new coordinates of each node of the distal face of the clot were computed in order to compute, for each node, the x, y, and z-displacements from the previous configuration. The total simulation time was chosen and divided by the number of centerline nodes, to obtain, for each node of the distal face of the clot, the three resulting time-displacement curves, which were then imported in LS-DYNA (Ansys, Canonsburg, PA, USA) as boundary conditions of the simulation.

A frictionless (to facilitate the movement of the clot inside the vessel) soft penalty contact between the clot and the vessel was activated. Once the clot was positioned, a soft penalty contact with static friction coefficient of 0.2 [52] was defined between clot and vessel.

The material model for rubber and foams based on a tabulated formulation of hyperelasticity proposed by Kolling et al. [53] and implemented in LS-DYNA was assigned to the clot. For its definition the user must specify a uniaxial stress–strain curve in tension and compression and the Poisson’s ratio. For each clot type (red, mixed, white) a different curve was loaded, while a Poisson’s ratio of 0.3 was set for all. The three curves and the Poisson’s ratio were obtained by testing human clot analogues of representative RBCs content for each clot type [54].

The simulations were run using the finite-element solver LS-DYNA R14 with 20 CPUs (Intel Xeon64) and 120 GB of RAM memory. A mass proportional damping was applied to the clot with coefficient of 0.1 ms^−1^ to improve stability. A selective mass scaling was used to use a constant timestep of 10^−3^ ms.

## Results

### Histological analysis

Table 3 reports the results of the histological analysis on the retrieved thrombi, along with the clot classification based on RBCs and fibrin content. Only one clot is classified as red (patient 3), three clots are classified as white (patients 1, 6, 7), and three are classified as mixed (patients 2, 4, 5).

**Table 3:** Results of the histological analysis on the retrieved thrombi in terms of RBCs, fibrin, and WBCs content, with the corresponding clot classification

Patient	RBCs content (%)	Fibrin content (%)	WBCs content (%)	Clot type
1	31.85	57.74	10.41	White
2	46.09	50.70	3.21	Mixed
3	62.46	33.22	4.32	Red
4	54.30	38.74	6.96	Mixed
5	41.08	52.47	6.45	Mixed
6	11.83	84.53	3.64	White
7	8.63	81.78	9.59	White

### Image analysis

**Fig. 4:**
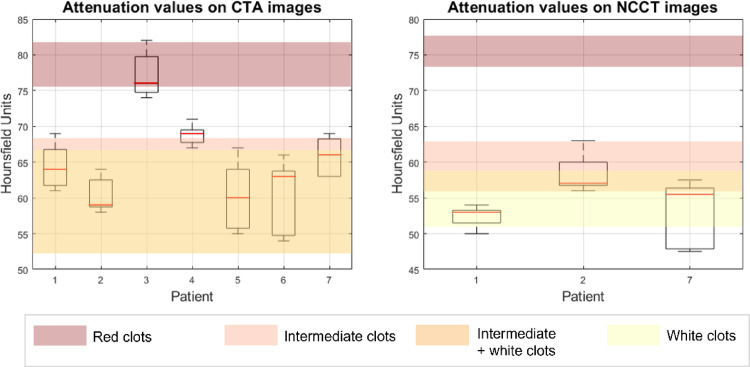
Box plots for the attenuation values measured in HU on the CTA images (left) and NCCT images (right). The red line indicates the median value, the lower end of the box is the 25th percentile, the upper end of the box is the 75th percentile, while the two whiskers are the minimum and the maximum values. The different colours identify the four regions corresponding to HU values of red, mixed and white clots and the overlapping region of mixed and white clots

Figure 4 shows the boxplots summarizing the measurements of HU on CTA and NCCT images. The median values were compared with the HU ranges reported in the work by Songsaeng et al. [45] for each clot type (values reported in Table 2), since, among the studies gathered in Table 2, it was the only one considering in vivo clinical images, and all NCCT, CECT, and CTA. Considering the CTA images, the median values for patients 1, 2, 5, 6, and 7 fell into the white-mixed thrombi range; the value for patient 4 was near the mixed thrombi range, while the value for patient 3 fell into the red thrombi range. Among the patients for whom a distinction between white and mixed was not possible on the CTA images, for patient 1, 2 and 7 the NCCTs were available. The median HU value for patients 1 and 7 fell into the white thrombi range, while the value for patient 2 fell again into the hybrid zone.

The results of the image analysis in terms of the position of the occlusion, estimated clot length, and the median HU on CTA and NCCT (if available) are reported in Table 4, along with the estimated clot type. For only one patient (patient 3) the measurement of the contrast gap was not possible, since the proximal end was not visible.

**Table 4:** Clot characteristics obtained from the analysis of radiological images

Patient	Position	Estimated clot length (mm)	Median HU from CTA	Median HU from NCCT	Estimated clot type
1	M1	13.5	64	53	White
2	MCA-bifurcation	9	59	57	White or mixed
3	T-junction	–	76	–	Red
4	M2	11	69	–	Mixed
5	T-junction	13	60	–	White or mixed
6	M2	6	63	–	White or mixed
7	M1	10.5	66	55.5	White

For each patient the position of the clot (confirmed by the clinical report) and the estimated clot length are reported along with the median attenuation value on CTA and NCCT (if available) and the consequent estimated clot type

Comparing the last column of Tables 3 and 4, it can be observed that for four out of seven patients (patients 1, 3, 4, and 7), the clot type estimated from the image analysis agreed with the classification based on histologic analysis. In the other three cases there was a partial agreement, since it was not possible to distinguish between white and mixed clots from the image analysis.

### FE simulation of clot positioning

**Fig. 5:**
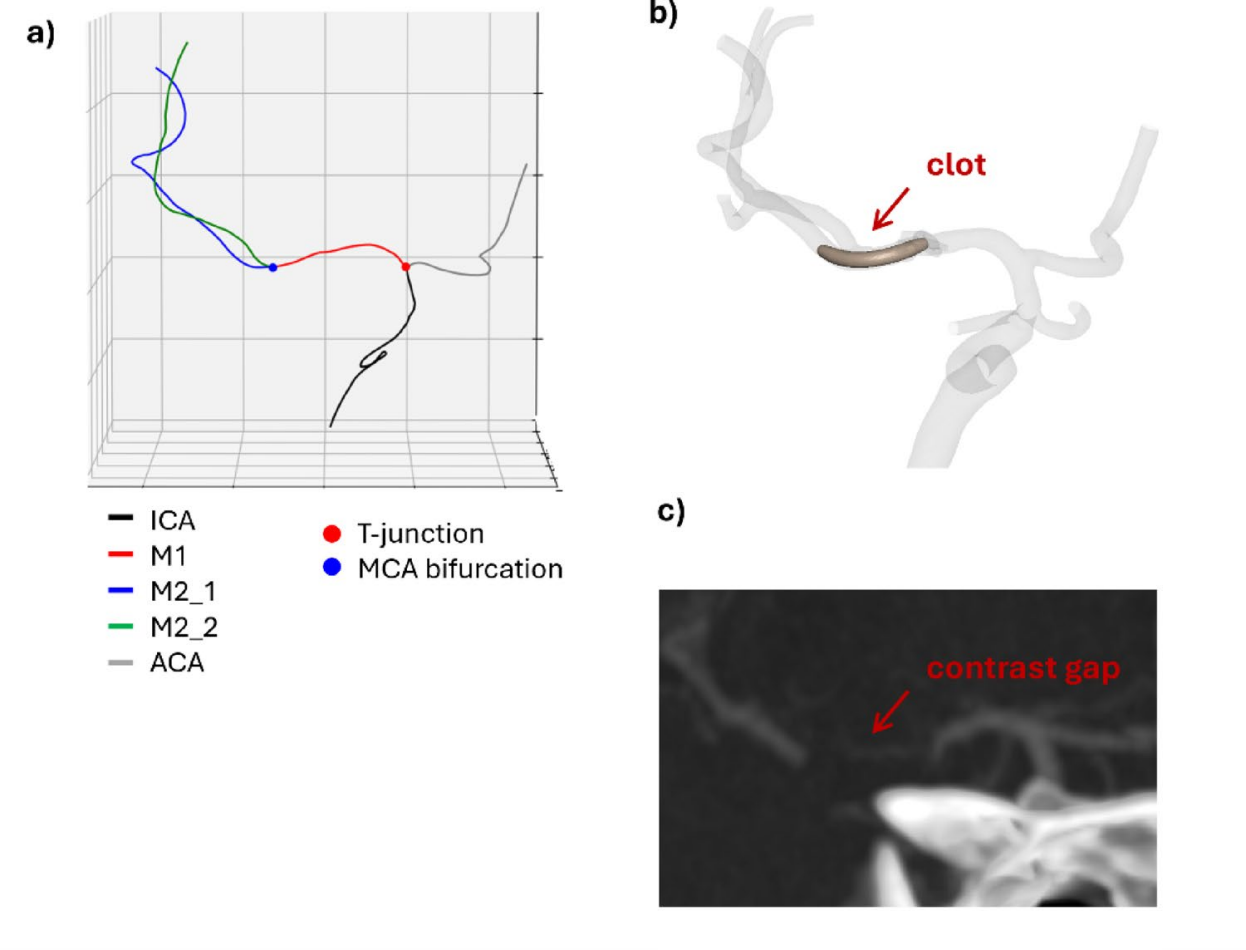
For one of the patients: **a** result of the Python script subdividing the centreline of the vessels in ICA, ACA, M1 and the two M2s with the identification of the T-junction (red dot) and the MCA bifurcation (blue dot); **b** final configuration obtained with the FE simulation; **c** CTA coronal view of the vessels of interest with visible contrast gap

Figure 5 shows the results of the identification of the different cerebral vessels using the first part of the Python code, along with the patient-specific model of the obstructed cerebral vessels obtained using the proposed methodology. In addition, the radiological image is shown for a qualitative comparison. For the patient shown in Fig. 5 a strong similarity can be observed, as for the other patients reported in Fig. S1.

## Discussion

Numerical simulations are, nowadays, widely employed for the study of clinical procedures, from the point of view of the performance of the devices and their interaction with the surrounding biological structures. To this purpose, our group developed a FE simulation of the MT procedure with stent-retriever [47], whose credibility was proved with an applicability analysis [48]. The methodology was then used to replicate a patient-specific case [49].

In this context, the present work aimed to define a methodology to create patient-specific models of AIS patients, recreating the condition before the MT procedure. The goal is to create a pipeline to guide the analysis of the available clinical data, to recreate the in vivo situation as accurately as possible. Firstly, information about clot position, approximate length, and type (i.e. composition) were obtained by examining radiological images. Secondly, two Python scripts were implemented to create the clot model and to compute the boundary conditions for the positioning FE simulation.

For five out of seven patients, the clot length was estimated from the measurement of the contrast gap on the CTA images. This measure was then refined with the help of the segmented 3D geometries. Using only the 3D geometries, by overlapping the 3D-CBCT pre and post intervention, does not add any information on the distal end of the contrast gap, since only the vessels up to the occlusion are reached by the contrast agent and therefore can be segmented in the pre-interventional 3D-CBCT. For patients 3 and 5 it was not possible to identify the proximal end of the contrast gap, thus for patient 3 an average clot length from the literature was assigned, while for patient 5 the measure was taken on the digital subtraction angiography (DSA). On this aspect, a first limitation must be mentioned: the contrast gap does not always coincide with the occluding clot. Indeed, depending on the position of the collateral vessels with respect to the occlusion, in some cases there could be a fraction of recirculating blood which is not reached by the contrast agent, both upstream and downstream of the clot. Thus, the contrast gap length should be considered an overestimation of the clot length. This is a limitation that must be considered when creating in silico models of AIS patients starting from CTA images.

Regarding the classification of clots into red, white and mixed based on their HU on the clinical images, a remark must be made about the literature data. The reference values in Table 2, show significant dissimilarities among the studies, particularly between the one of Songsaeng et al. [45] and the pair Cruts et al. [42] and Ding et al. [41]. In contrast to Songsaeng et al. [45] which measured the HU values on different clinical images of AIS patients, the works by Cruts et al. [42] and Ding et al. [41] were in vitro studies using clot analogues. They reported HU values for white clots [41, 42] and mixed and red clots [41] which were substantially lower than the ones reported by Songsaeng et al. [45] for the same imaging modality (NCCT). The reason behind this difference may be in the lower x-ray attenuation of the fluid used in the in vitro experiments (Dulbecco’s Modified Eagle Medium for Cruts et al. [42] and water for Ding et al. [41]) with respect to the surrounding in vivo tissues. For this reason, considering the HU ranges proposed by Ding et al. [41], clots of patients 1, 2, and 7 would have been classified as red contrary to their classification as white, mixed and white, respectively, based on histologic analysis. Another factor that could explain the low HU values reported for white clots by Cruts et al. [42] is that the only white clot composition considered was 100% fibrin.

Since the NCCTs were not available for all patients, the HU values were measured on the CTA images and then compared with the ranges reported by Songsaeng et al. [45]. The HU ranges for white and mixed clots almost completely overlap when CTA images were considered. Thus, in the present work, it was possible to discern only red clots from non-red clots (mixed and white). Further discerning between white and mixed clots was possible in only two cases (patients 1 and 7) since the median HU value on NCCT images fell in the white range reported by Songsaeng et al. [45].

For four out of seven patients, the clot type estimated from the images was fully consistent with the clot type identified by histological analysis, using the classification suggested by Songsaeng et al. [45] based on RBCs and fibrin percentual contents. In the other three cases, there was only partial agreement since from the images it was not possible to distinguish between white and mixed clots due to the limitations of the literature data mentioned above.

Starting from the reference clot geometry, two Python scripts were implemented to scale and discretize the clot geometry and to compute the boundary conditions for the simulation of clot positioning at the occlusion site. The advantage of this methodology is that once the reference clot geometry is created (once and for all) and the user selects the initial and final points on the centerline, the setup of the simulation is automatic. Then, the simulation of clot positioning took a maximum of 5 min to run. In these simulations, the different clot material models (white, mixed, red) were assigned according to the classification based on histological analysis. However, in the future, this assignment should be based on image analysis classification.

Finally, the qualitative comparison between the simulation outcomes and the clinical images showed a strong similarity between the two, proving the developed methodology to be able to reproduce a variety of patient-specific cases with occlusion in different positions and with any clot dimensions, in a time-efficient manner. This is a fundamental step in the in silico replica of patient-specific MT cases.

The present work is not free from limitations. The first limitation in the analysis of the data is given by their heterogeneity among patients because patients can come from multiple primary stroke centers and due to the urgent need for intervention in case of AIS symptoms and after the AIS diagnosis. Then, in the positioning FE simulation (i) the vessel was modeled as rigid, an assumption often made for MT simulations in the literature, and, (ii) a translational boundary condition was imposed on the distal end of the clot for the positioning, while a more realistic positioning would include the application of a pressure to the proximal face of the clot or the inclusion of a moving fluid to push the clot, which would, however, result in a more complex simulation with a less controllable clot positioning.

## Conclusions

In this work, routine clinical images for the AIS diagnosis were analyzed to extract patient-specific information on clot position, length, and, where possible, composition. First, the contrast gap was measured on CTA images to estimate the clot length. Second, clot composition was inferred based on the HU measured in the contrast gap: using literature-based HU ranges, it was possible to distinguish RBC-rich (red) and RBC-poor (white and mixed), without further distinguishing RBC-poor clots into white and mixed. These findings confirm and highlight the difficulties of determining clot composition prior to the MT intervention, except for red hyperdense clots, which are typically identifiable. Indeed, to assign appropriate clot mechanical properties in the FE simulation, histological analyses were used to classify the clots into red, white, and mixed. Then, two Python scripts were developed to semi-automatically set up the FE simulation of clot positioning, to build the AIS patient-specific model. For each patient, the simulated clot position qualitatively reflected the occlusion observed in the clinical images, making these patient-specific models the starting point for patient-specific simulations of the entire MT procedure.

## Supplementary Information

Below is the link to the electronic supplementary material.

Supplementary Material 1
