# Supplementary material for: Clinical image analysis to build patient-specific models of acute ischemic stroke patients
Source: Phys Eng Sci Med. 2025 Sep 29;49(1):27–37. doi: 10.1007/s13246-025-01646-7 (PMC12987914; doi:10.1007/s13246-025-01646-7)
Supplement: Supplementary file 1 — Supplementary Material 1 [file 13246_2025_1646_MOESM1_ESM.docx]

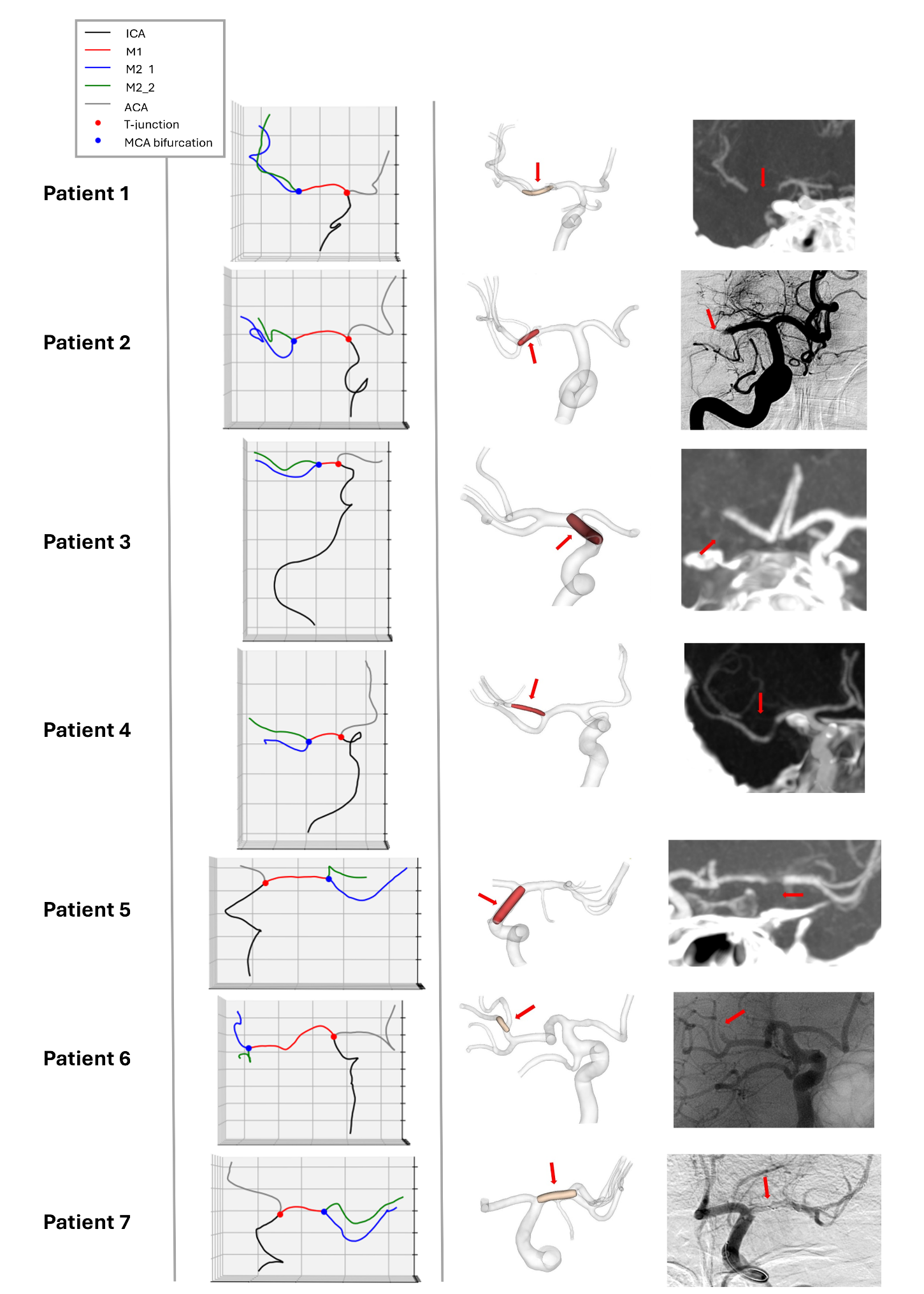


Figure S1 On the left: result of the Python script subdividing the centreline of the vessels in ICA, ACA, M1 and the two M2s with the identification of the T-junction and the MCA bifurcation. On the right: comparison between the final configuration obtained with the FE simulation and the clinical images.
